# Photocatalytic Degradation of 4,4′-Isopropylidenebis(2,6-dibromophenol) on Magnetite Catalysts vs. Ozonolysis Method: Process Efficiency and Toxicity Assessment of Disinfection By-Products

**DOI:** 10.3390/ijms23073438

**Published:** 2022-03-22

**Authors:** Joanna Kisała, Anna Tomaszewska, Adriana Barylyak, Yaroslav Bobitski, Maciej Balawejder

**Affiliations:** 1 Department of Biology, Institute of Biology and Biotechnology, University of Rzeszow, Pigonia 1, 35-310 Rzeszow, Poland; 2 Department of Biotechnology, Institute of Biology and Biotechnology, University of Rzeszow, Pigonia 1, 35-310 Rzeszow, Poland; atomaszewska@ur.edu.pl; 3 Department of Therapeutic Dentistry, Danylo Halitsky Lviv National Medicinal University, Pekarska Str. 69, 79010 Lviv, Ukraine; adriana.barylyak5@gmail.com; 4 Department of Photonics, Lviv Politechnic National University, 1 Sviatoho Yura Sq., 79013 Lviv, Ukraine; yaroslav.v.bobytskyi@lpnu.ua; 5 Center for Microelectronics and Nanotechnology, Institute of Physics, University of Rzeszow, Pigonia 1, 35-959 Rzeszow, Poland; 6 Department of Chemistry and Food Toxicology, Institute of Food Technology and Nutrition, University of Rzeszow, St. Cwiklinskiej 1a, 35-601 Rzeszow, Poland; mbalawejder@ur.edu.pl

**Keywords:** TBBPA degradation, ozonolysis, photocatalysis, COD, TOC, magnetite, microbiotest

## Abstract

Flame retardants have attracted growing environmental concern. Recently, an increasing number of studies have been conducted worldwide to investigate flame-retardant sources, environmental distribution, living organisms’ exposure, and toxicity. The presented studies include the degradation of 4,4′-isopropylidenebis(2,6-dibromophenol) (TBBPA) by ozonolysis and photocatalysis. In the photocatalytic process, nano- and micro-magnetite (n-Fe_3_O_4_ and μ-Fe_3_O_4_) are used as a catalyst. Monitoring of TBBPA decay in the photocatalysis and ozonolysis showed photocatalysis to be more effective. Significant removal of TBBPA was achieved within 10 min in photocatalysis (*ca.* 90%), while for ozonation, a comparable effect was observed within 70 min. To determine the best method of TBBPA degradation concentration on COD and TOC, the removals were examined. The highest oxidation state was obtained for photocatalysis on μ-Fe_3_O_4_, whereas for n-Fe_3_O_4_ and ozonolysis, the COD/TOC ratio was lower. Acute toxicity results show noticeable differences in the toxicity of TBBPA and its degradation products to *Artemia franciscana* and *Thamnocephalus platyurus*. The EC_50_ values indicate that TBBPA degradation products were toxic to harmful, whereas the TBPPA and post-reaction mixtures were toxic to the invertebrate species tested. The best efficiency in the removal and degradation of TBBPA was in the photocatalysis process on μ-Fe_3_O_4_ (reaction system 1). The examined crustaceans can be used as a sensitive test for acute toxicity evaluation.

## 1. Introduction

Brominated flame retardants (BFRs) are found in many household, health care, and consumer products, representing 25% of the global market of flame-retardant compounds. Due to its fireproof properties, it is widely used in everyday products such as furniture, upholstery, adhesives, and electronic equipment. A widespread use of TBBPA can contribute to environmental pollution. TBBPA has been found in soil, water, river sediments, and the atmosphere. This compound is characterized by a high value of log P_oct/water_ = 4.75 (pH = 7.5) and a low acidity. It may exist in undissociated or dissociated form, depending on the pH of the system (pK_a1_ = 7.5; pK_a2_ = 8.5). Due to the high hydrophobicity, TBBPA may accumulate in living organisms at different food chain levels. Although TBBPA is not classified as acutely toxic, it has been shown to elicit various toxic responses in the aquatic environment [1]. Its molecular structure is analogical to thyroxine (thyroid hormone). In vitro tests have shown a strong chemical affinity of TBBPA for transthyretin protein (TTR), which may cause disturbances in the organism’s hormonal balance. Recent studies indicate that TBBPA can cause several diseases, such as cancer, obesity, anomalies in the immune system, and reproductive capacity impairment.

The common existence (even in household dust) and the risky transformation of TBBPA and BPA in the environment have attracted the attention of researchers around the world in recent years [2,3]. The study of TBBPA degradation processes and the toxicity of the by-products is extremely important because of its highest production volume out of all BFRs in the world. The TBBPA market size is forecasted to reach $3.5 billion by 2025 [4]. Under natural conditions, the degradation of TBBPA seems to be problematic. Water and soil contamination removal techniques usually require complex procedures, whereas thermal treatment is a classic approach to TBBPA decomposition [5]. One of the major drawbacks of TBBPA combustion or pyrolysis relies on highly toxic PBDDs (dibromodichlorodibenzo-p-dioxins) and PBDFs (dibromodichlorodibenzofurans) [6]. In contrast, advanced oxidation processes, such as ozonolysis or photocatalysis, have been considered as a promising technique for TBBPA degradation, free of the above-mentioned drawbacks. Ozonolysis leads to the almost complete removal of bromine from TBBPA and the formation of BPA. This process may occur directly in the atmosphere and water solutions [7]. Unfortunately, the formation of BPA during the process is a serious disadvantage. BPA is a genuine threat to living species, not only due to their estrogenic activity, but also because of adverse effects such as genotoxicity and carcinogenicity. Whereas the photocatalysis process causes disruption of TBBPA and the formation of small-molecule compounds.

Bioassay techniques have been the basis of environmental security and chemical safety programs [8]. Over the last decade, the uses of aquatic bioassays have been extended from water pollution control to the determination of potential toxins to aquatic life. Various assays are used for research of the potential toxicity of persistent organic pollutants (POP’s) based on different biological models, such as in vivo assays on laboratory animals. However, recent studies have employed efforts for alternative biological assays that include species of *Artemia salina*, *Artemia franciscana*, *Artemia urmiana,* and *Thamnocephalus platyurus*. These toxicity tests are considered a useful tool for the preliminary assessment of toxicity [9,10,11].

This work investigates the efficiency of AOPs (photocatalysis on Fe_3_O_4_ catalysts, and ozonolysis) in TBBPA removal from water and the impact of pre-treated water solutions on selected model organisms (saltwater crustaceans and freshwater crustaceans). The purpose of this study was to measure the toxicities (as 24-h EC_50_) of TBBPA, its degradation products (pure substances), and pre-treated TBBPA solutions by ozonolysis or photocatalytic processes (post-reaction mixtures) to *T. platyurus* and *A. franciskana*. The obtained results showed that, in the case of freshwater crustaceae, the resulting products have a higher toxicity than the starting compound. In contrast, saltwater organisms were more resistant.

## 2. Results and Discussion

**Figure 1:**
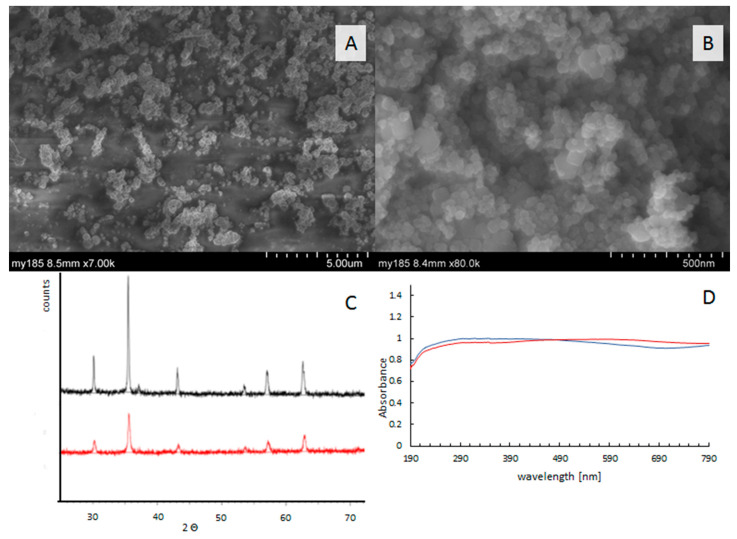
The catalysts’ characterization. SEM images of Fe_3_O_4_ micro-size (**A**) and nano-size (**B**). X-ray diffraction measurements of μ-Fe_3_O_4_ (black), n-Fe_3_O_4_ (red) and (**C**), UV−VIS absorption spectra of μ-Fe_3_O_4_ (red) and n-Fe_3_O_4_ (blue) (**D**).

The morphology of Fe_3_O_4_ catalysts is shown in Figure 1A,B. Images show agglomerates of nanocrystals with particle sizes between 100–400 nm (Figure 1A) for micro-catalysts, and 25–100 nm (Figure 1B). XRD patterns showed that the diffraction peaks surrounding 2θ = 30.2°, 35.3°, 43.7°, 53.9°, 57.1°, and 62.7° (Figure 1C) were in good agreement with Fe_3_O_4_ (reference code 01-089-3854); they belonged to the cubic structure system, corresponding to (220), (311), (400), (422), (511), and (440) facets of Fe_3_O_4_, respectively [12]. The absorption spectra in the range of 180–1000 nm are similar for both catalysts and show that catalysts effectively absorb irradiation from the visible range (Figure 1D). The phase identification of the magnetite structure was carried out with a powder X-ray diffractometer (Figure 1D).

**Figure 2:**
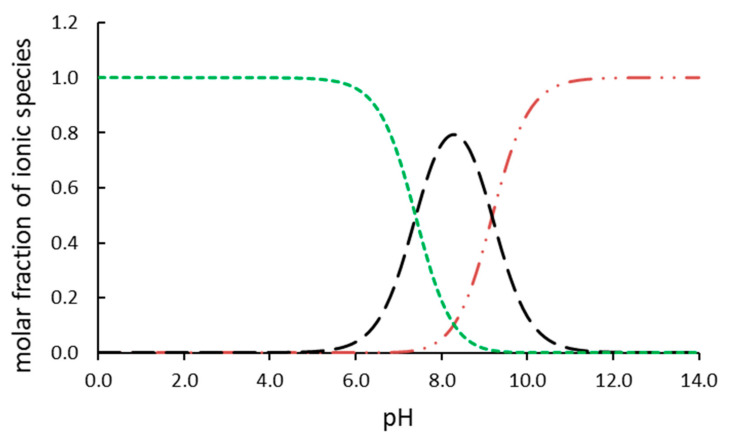
Ionic species of TBBPA in solution: TBBPA (green), TBBPA^–^ (black), and TBBPA^2–^ (red).

**Figure 3:**
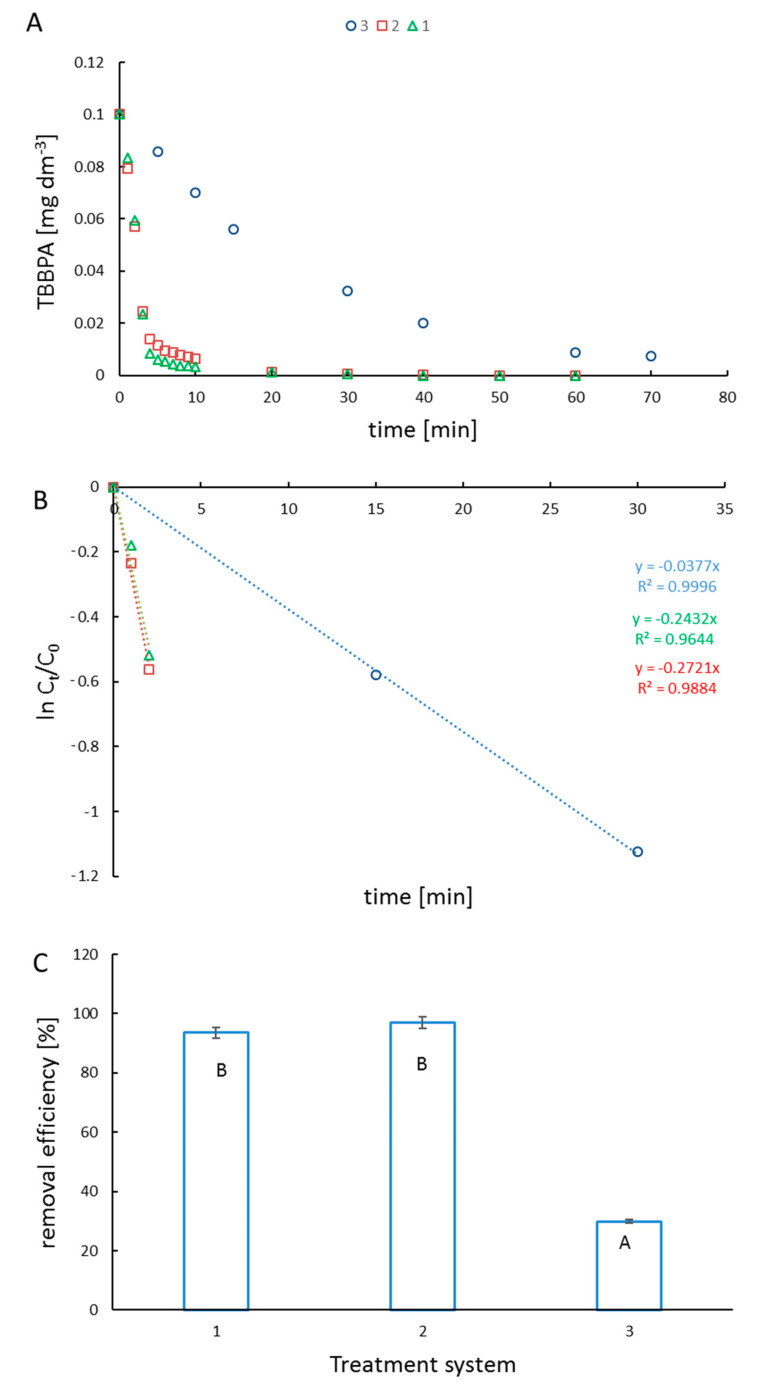
TBBPA decay rate in different degradation systems (**A**) (treatment system 1—triangles, 2—squares, 3—circles); plot of ln (C_t_/C_0_) vs. irradiation time (**B**); removal efficiency in used degradation systems (description in Table 1) (**C**). On the figure removal efficiency ± SD, n = 3 was given, statistical (*p* < 0.01) differences between the degradation methods, assessed by Statistica 13.1 Student’s test, are indicated by different letters.

In our study, TBBPA was removed from the reaction mixtures in two different processes listed in Table 1. The reactions were conducted in aqueous solutions at pH = 8. At these conditions, TBBPA exists in three different forms, including the molecular form and two dissociated forms. The simulated (using pK*_a_* of compounds and Curtipot software [13]) percentage molar fraction of ionic forms at pH = 8 is: TBBPA—20%, TBBPA^−^—75%, and TBBPA^2−^—5% (Figure 2). The decay of TBBPA in the time suggests that the ionic composition of the substrate improves the photocatalytic degradation, whereas it hinders the reaction with hydroxyl radicals. The removal efficiency (Figure 3B) shows the excellent effectiveness of photocatalysis compared to ozonolysis.

The ozone in water (pH > 7) reacts with a hydroxyl anion yielding hydroxyl radicals, thus the **·**OH radical dominates in the reaction system 1 as an oxidizing agent.

O_3_ + ¯OH → **·**OH (1)

**·**OH + S→ **·**R (2)

Therefore, the degradation of TBBPA occurred with **·**OH radicals, while molecular ozone may be ignored.

In the photocatalytic process, nano- and micro-magnetite were used as a catalyst. Iron oxides, apart from their high catalytic activity, are ecological, cheap, and easily available. Magnetite is a common material found in igneous, metamorphic, and sedimentary rocks. Due to its universal nature, it can take part in changes in organic pollutants in the environment. Hence, the photocatalytic system proposed by the authors did not require high operating costs: it comprises the commonly available iron (II, III) oxide (magnetite) and uses visible radiation.

The magnetite surface in the aqueous media possesses acid−base properties with pK values of 4.4 (pK_1_) and 9.0 (pK_2_) [14]. At acidic conditions, Fe_3_O_4_ dissolves, whereas, at pH > 7 the effect of hydrolysis is expected to be negligible [15]. Monitoring the TBBPA decay in the photocatalysis and ozonolysis showed photocatalysis to be more effective (Figure 3A). Significant removal of TBBPA was achieved within 10 min in photocatalysis, while for ozonation, a comparable effect was observed within 70 min. The observed removal efficiency (Figure 3C) for catalysts is comparable. The ozonolysis method of TBBPA removal is relatively low.

Catalytic experiments were carried out in three reaction systems in order to investigate the best degradation effect. The photodegradation rate constant (k_app_) was determined for each degradation system, with the assumption that the ongoing reactions were of the first order from Equation (3):

ln (C_t_/C_0_) = −k_app_ t (3)

where k_app_ is the apparent rate constant, and C_0_ and C_t_ are the initial concentration and concentration at time t, respectively.

The plot of ln C_t_/C_0_ versus time represents a straight line, as shown in Figure 3B. The TBBPA degradation kinetics concluded the pseudo-first-order kinetics (R^2^ > 0.95). The kinetic results proved that the photocatalytic process was very effective through degradation by ozonolysis.

**Figure 4:**
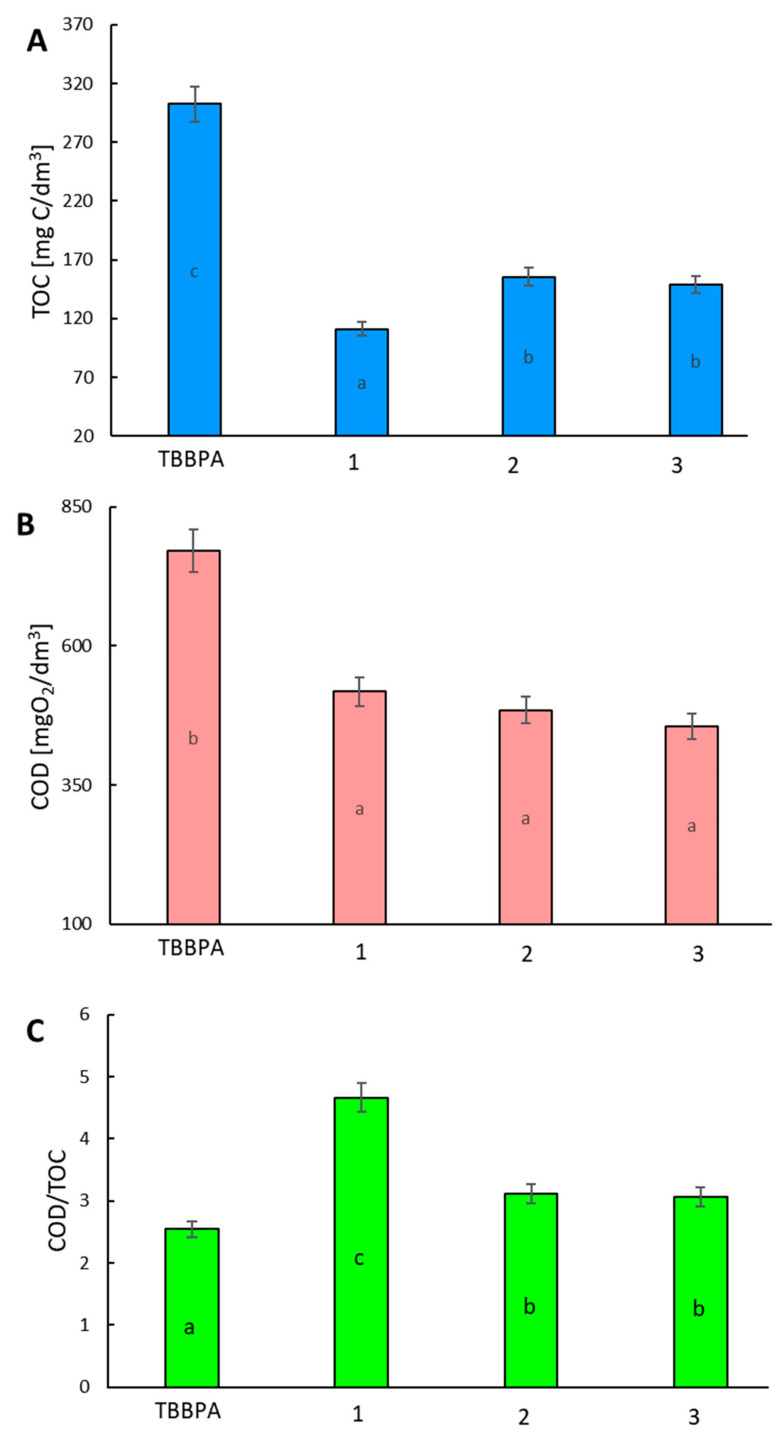
Concentration of organic compounds in solutions of raw TBBPA and products of AOP treatment expressed as a total organic carbon (TOC) (**A**), chemical oxygen demand (COD) (**B**), and TOC/COD ratio (**C**). The mean value ± SD, n = 3 of the result was presented, statistical (*p* < 0.01) differences between the results, assessed by Statistica 13.1 Student’s test, are indicated by different letters.

The total organic carbon (TOC) concentration in the samples is widely used as an indicator of organic carbon behavior when evaluating water pollution, soil and sediment organic matter, and the carbon cycle [16,17,18,19]. In particular, TOC has attracted attention as an indicator of non-biodegradable organic matter, including particulate pollutants from various non-point sources; it is considered instead of the chemical oxygen demand (COD) test [20,21,22,23]. To determine the best method of TBBPA, the degradation concentration of COD and TOC removal were examined. The COD/TOC ratio also yields interesting information on how chemical substances become more oxidized, with lower ratios meaning higher oxidation. The reduction of the COD/TOC ratio is related to the formation of difficult to oxidize intermediates. It was found that the initial value of the TOC parameter for the TBBPA stock solution was 302.52 mg dm^−3^. The AOP pre-treatment caused a decrease in TOC concentration. The TOC removal efficiency was comparable for each of the pre-treatment methods (ranging between 63–48%). The degradation system influenced the TOC removal efficiency (the highest for 1, and the lowest for 3 (Figure 4A)). The lower TOC removal efficiency in system 2 may be involved with the surface properties of the catalyst and pK_a_ of organic compounds. The AOP pre-treatment resulted in a significant decrease in COD value in all of the experimental layouts (Figure 4B). The COD/TOC value determines the amount of oxygen that is needed to oxidize organic substances in relation to the carbon content in their chemical structure. The value of the COD/TOC ratio depends on the structure of the organic compounds present in the solution, including the oxygen content in the molecule. In the present work, the COD/TOC ratio for TBBPA was 2.55. After AOPs pre-treatment, this proportion increased in all reaction systems (Figure 4C). Hence, the reaction system **1** caused a rise in oxidized compounds.

**Figure 5:**
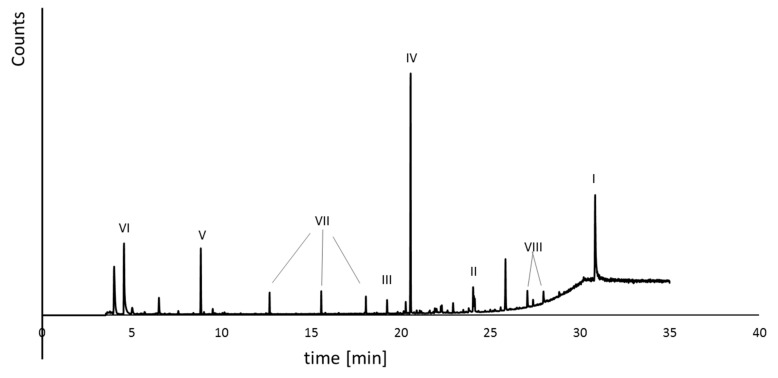
Exemplary GC-MS analysis of the reaction mixture, where I—TBBPA; II—BPA; III—3,5-dibromo-4-hydroxybenzoic acid; IV—2,6-dibromo-4-isopropylphenol; V—2,6-dibromo-4-methylphenol; VI—DMSO; VII—aliphatic carboxylic acids; VIII—tri-, di-, mono-bromobisphenol A.

The formed by-products were qualitatively estimated by GC-MS analysis (Figure 5). The reaction mixtures contained the following degradation products: BPA (debromination process), 2,6-dibromo-4-isopropylphenol (cleavage of molecules), and other aromatic compounds (e.g., 3,5-dibromo-4-hydroxybenzoic acid, 2,6-dibromo-4-methylphenol, bromophenol, phenol; see Figure 5). The toxicity tests were conducted using pure for the analysis of the compounds and reaction mixture.

**Figure 6:**
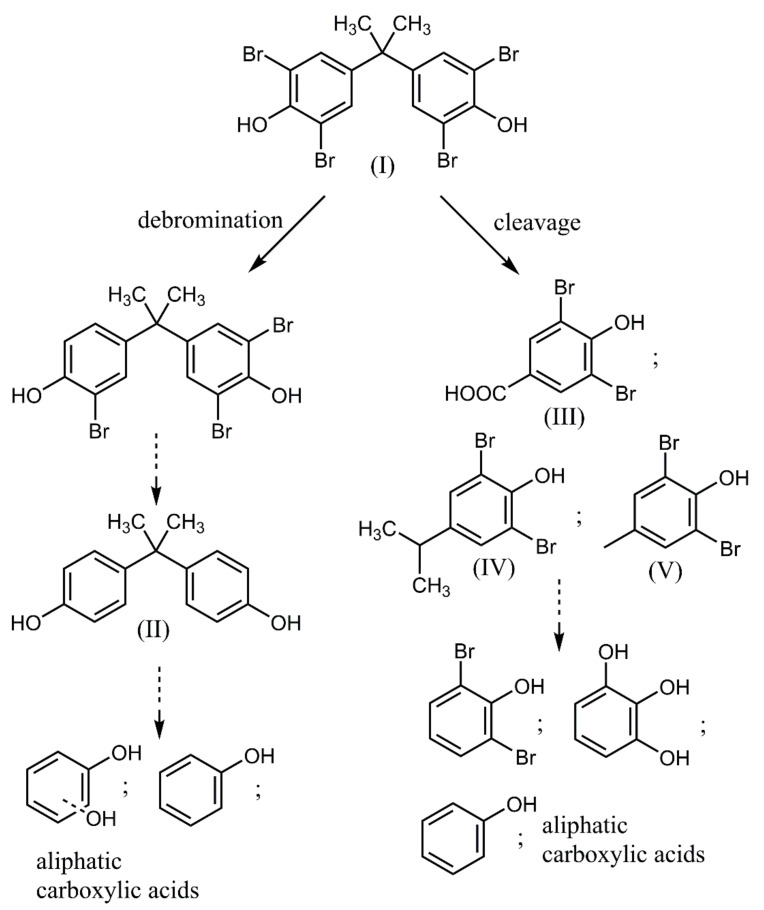
Schematic presentation of TBBPA degradation organic products.

Direct photolysis of halogenated aromatics in water usually proceeds through dehalogenation [24], although subsequent system changes depend on the position of the substituents. In the case of photocatalytic degradation of BPA and TBBPA, the primary reaction involves cleavage between one of the benzene rings and the isopropyl group. TBBPA degradation mainly involves de-bromination, hydroxylation, and demethylation in both oxidation and reduction processes [25]. The intermediate products of BPA/TBBPA decomposition during photocatalytic degradation include low molecular fraction formation and its subsequent mineralization (Figure 6). The intermediate products of BPA decomposition during photocatalytic degradation include p-isopropenylphenol, hydroquinone, 4-isopropenylphenol, glycolic acid acetate, tartaric acid, and formic acid [26,27]. Aliphatic acids as intermediates are formed by the further oxidation of quinone derivatives [28]. It is known that phenol is photocatalytically mineralized via the generation of quinone derivatives and organic acids as the intermediates.

Because of the low solubility of TBBPA, degradation processes and toxicological studies of TBBPA were conducted with co-solvent DMSO. TBBPA is an excellent soluble in methanol, acetone, and dimethyl sulfoxide (DMSO) [29]. Dimethyl sulfoxide has been chosen due to its low toxicity for the tested crustacea (up to 11% concentrations, [30]). The percentage of DMSO in each solution was 0.1% (*v*/*v*).

Due to its widespread distribution, TBBPA inevitably makes its way into aquatic environments such as rivers, lakes, and estuaries. Its concentration in the surface waters reached 4.87 mg dm^−3^ in Eastern and Southern China [31], a high concentration of TBBPA was detected in the Detroit River in the USA (0.6–1.84 μg dm^−3^) [32], whereas in freshwater sediments from the River Skerne in northeast England 9.8 mg kg^−1^ dry weight was detected [33]. Based on these data, the concentration of TBBPA for acute toxicity studies was established at 0.1 mg dm^−3^. The BPA concentration was used analogous to Castritsi-Catharios et al. [34]. Thus, we chose the following concentrations for the toxicity tests of the organic compounds: TBBPA 0.1 mg dm^−3^, BPA 55 mg dm^−3^, 2,6-dibromo-4-isopropylphenol 50 mg dm^−3^, 2,6-dibromo-4-methylphenol 51.65 mg dm^−3^, and 3,5-dibromo-4-hydroxybenzoic acid 52 mg dm^−3^. The values of EC_50_ (24 h, immobility) of organic compounds and reaction mixtures to *A. franciscana* and *T. platyurus* were estimated using nominal concentrations. The values were calculated using a Regtox spreadsheet [35] supplied with the microbiotest. The results of all toxicity studies were acceptable, because the control mortality did not exceed 10%.

**Figure 7:**
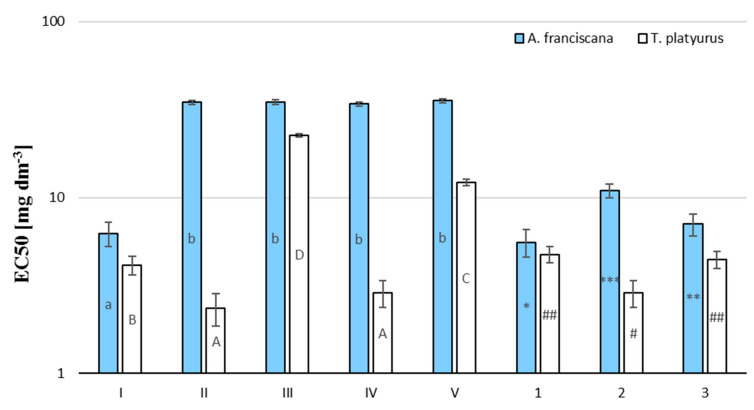
Mean 24 h EC_50_ values of the investigated compounds and post-reaction mixtures for A. franciscana (blue) and T. platyurus (white). The mean value ± SD, n = 3 of the result was presented, statistical (*p* < 0.01) differences between the results, assessed by Statistica 13.1 Student’s test, are indicated by different small letters for A. franciscana, capital letters for T. platyurus, number of *, **, *** for post-reaction mixtures tested on A. franciscana, number of ^#^, ^##^ for post-reaction mixtures tested on T. platyurus, difference at *p* < 0.05.

Acute toxic values were expressed as EC_50_ values (concentration for 50% of maximal effect, mg/L) with 95% confidence intervals and were calculated by the Regtox spreadsheet. The obtained values of EC_50_ 24 h are summarized in Table 2 and are presented visually in Figure 7.

Brine shrimp and freshwater shrimp bioassays are considered as a rapid preliminary screening for biochemical activity, and were used to determine the selected phenolic’s compound toxicity. These tests are based on the potential of the compound to become lethal to *A. franciscana* and *T. platyurus* nauplii due to its toxic expression. Among the pure substances tested by us, the most toxic appeared TBBPA (toxic in European Union Commission classification, Table 3). Compounds II and V show the same toxicity to both of the tested organisms, set at the harmful level. The reaction mixtures after the degradation processes are as toxic as the starting material. A reduction in toxicity was only observed in the cause n-Fe_3_O_4_ photocatalysis for *A. franciscana*.

The degradation of TBBPA solution in different conditions negligibly affected the toxicity of the reaction mixture compared to pure TBBPA. Pure compounds: (II), (III), (IV), and (V) were also found to be toxic and harmful to *A. franciscana*. Their EC_50_ was higher than for TBBPA (Table 2). The tested organic compounds exhibited a slightly higher toxic potency to *T. platyurus* than *A. franciscana*, the exception was compounds (II) and (IV), which turned out to be *ca.* 10-fold more toxic to *T. platyurus* than *A. franciscana*. Photodegradation of TBBPA on n-Fe_3_O_4_ causes a slight decrease (ca. 2-fold) in the post-reaction mixtures’ toxicity compared to that of the original TBBPA (Figure 6). In the case of the TBBPA degradation on nano Fe_3_O_4_, a decrease in toxicity towards *A. franciscana* (1.75 times) and an increase in the mixture’s toxicity after treatment towards *T. platyurus* (1.43 times) were observed. The higher resistance of A. franciscana may result from the conditions in which this crustacean occurs naturally (water with a salinity up to 200%). Comparing the EC_50_ values of pure compounds and post-reaction mixtures, it can be concluded that the post-reaction mixtures are characterized by a higher toxicity than the toxicity of the by-products formed during the ozonolysis/photocatalysis processes.

It should be noted that the correlation between the results of the Artoxkit:Thamnotoxkit tests determined for pure compounds (Pearson correlation coefficient r = 0.49) and post-reaction mixtures (Pearson correlation coefficient = −0.99) was recognized.

Bisphenol A toxicity has been studied extensively in the literature. Economou et al. [37] found that the mean values of LC_50_ were 45.51 mg dm^−3^ for *A. franciscana*. The LC_50_, EC_50_, and IC_50_ values of BPA reported previously for other aquatic organisms vary between 4.6 to 17.93 mg dm^−3^ for fish and 0.3 to 10 mg dm^−3^ for invertebrate species [38,39,40]. The LC_50_ values estimated by Catritsi et al. [34] after exposure for 24 h were 44.8 (44.6–45) mg dm^−3^. In our research, EC_50_ for *Artemia* nauplii after 24 h exposure was 44.8 (44.6–45.00) mg dm^−3^. Debenest et al. [41] estimated the mean values of TBBPA LC_50_ for *T. platyurus* as 8.3 (7.1–9.6) mg dm^−3^. The differences in EC_50_ values reported by other researchers and determined by us may be because of several factors. First, during the degradation process, the bromine atoms in aromatic rings were released as bromine (Br^−^), which could be subsequently oxidized to bromate (BrO_3_^−^). Notably, BrO_3_^−^ is a category I group B2 carcinogen. Molecular TBBPA could be more toxic to crustacea’s than dissociated forms, because it can interact with phospholipid membranes to distribute throughout all regions of the phospholipid bilayer to influence biological-involving cell membranes [42]. However, no apparent impact of DMSO alone on the examined crustacea was found. DMSO is a lipophilic molecule and is thought to be relatively permeable throughout the cell membrane [43]. In this regard, DMSO could allow the TBBPA molecule to enter to the cell more easily. This may explain the lower value of EC_50_ for TBBPA obtained by us than that mentioned above. The difference in LC_50_ value for *Artemia* nauplii could be possibly attributed to adaptive mechanisms and higher resilience in environmental pressure.

The results obtained in the present study indicate that the acute toxicity test is sufficient to confirm the effectiveness of wastewater detoxification in the wastewater treatment process. Research on the removal of organic micro-pollutants from water usually focuses on the disappearance of a chemical compound in the system, without examining the toxicity of the treated solution. The aim of water treatment should not be the disappearance of organic pollution, but the absence of toxicity of the purified sample. The tests performed have shown that toxicity tests are a useful tool for checking effluent detoxification. The selected model organisms showed that both the micropollutants study and their degradation products are bioavailable. Toxicity tests measure the aggregate effects of contaminated media on organisms (e.g., ionic strength, interactions among contaminants, and interactions between contaminants and media). Two types of crustaceans were selected for toxicity tests for this reason: high salinity resistant (*A. franciscana*) and more sensitive freshwater (*T. platyurus*). Current studies have shown that acute toxicity tests using crustaceans are a useful tool for rapidly assessing the toxicity of the treated solutions. What is more, we have shown that removing the organic contaminants from a solution does not always result in a lack of toxicity.

## 3. Materials and Methods

### 3.1. Chemical Reagents and Catalysts

4,4′-Isopropylidenebis(2,6-dibromophenol) (97%) was purchased from Alfa Aesar (Haverhill, Massachusetts, USA), Bisphenol-A (BPA) (>98%) from Sigma-Aldrich (Saint Louis, Missouri, USA). The catalysts were obtained from Sigma-Aldrich: Fe_3_O_4_ magnetite micropowder (μ-Fe_3_O_4_), particle size under 5 μm, d = 4.8–5.1 g cm^−3^, Fe_3_O_4_ nanopowder (n-Fe_3_O_4_), particle size under 50 nm (derived from TEM), d = 3.9 g cm^−3^, >98% trace metals. All of the chemicals were of analytical grade and were used without further purification if there was no special explanation.

### 3.2. Catalyst Characterization

The crystal structure of the materials was studied using X-ray diffraction (XRD) (D8 Advance, BRUKER, Billerica, Massachusetts, USA) with a graphite monochromator using CuK_α_ radiation (λ = 1.5406 A), within 20–100 2 θ. The catalyst morphology was observed using a SU-8010 scanning electron microscope (SEM) (Hitachi, Tokyo, Japan). The catalysts’ optical properties were investigated using the Cary series UV−VIS-NIR spectrophotometer (Agilent Technology, Santa Clara, California,, USA) in the range of 180–1200 nm.

### 3.3. Degradation Procedure

#### 3.3.1. Photocatalytic Degradation of TBBPA

In a typical experiment, 750 cm^3^ of 0.1 g dm^−3^ (1.84 × 10^−4^ mol dm^−3^) TBBPA solution (in all cases a low water-solubility of TBBPA enforced the usage of DMSO as a co-solvent in concentrations of *ca.* 0.1% *v*/*v*), pH adjusted using 0.1 mol dm^−3^ NaOH solution, to a value of 8 ± 0.1, being within the pH range of the magnetite stability, and next 0.07% of the catalyst powder was dispersed. Then, the obtained suspension was stirred for 30 min in the dark and was atmospheric air-saturated. The photocatalytic degradation was performed using a Heraeus LRS2 glass photoreactor (of 750 cm^3^ volume). The illumination was affected with the excimer lamp TQ150 (150 Watt, with forced water cooling down to 25 °C, of *ca.* 47 W light energy flux integrated over the 200–600 nm range, of power density 0.004696 W cm^−2^ measured by digital lux meter Peak Tech 5025 what gives light intensity *ca.* 7.88 × 10^19^ photons per second) operated by utilizing a vertically arranged immersion tube, immersed into the continuously stirred reaction suspension. The photocatalytic reaction was performed up to 60 min.

#### 3.3.2. Ozonolysis

The ozonolysis experiment was performed as follows: 750 cm^3^ of 0.1 g cm^3^ (1.84 × 10^−4^ mol dm^−3^) TBBPA solution (with DMSO as the co-solvent in the concentrations of *ca.* 0.1% *v*/*v*) and the pH was adjusted using 0.1 mol dm^−3^ NaOH solution, to a value of 8 ± 0.1. The reaction was developed using the Viaken Vairo-2186 ozone generator (Viaken, Krakow, Poland). The ozone concentration in the gas phase, measured following the iodometric method [44], was 12.73 mg dm^−3^h^−1^. The ozonolysis reaction was performed up to 70 min.

Post reaction mixtures were examined for organic carbon forms’ concentration, expressed as COD and TOC [45,46].

#### 3.3.3. Toxicity Tests

The Thamnotoxkit F™ and Artoxkit M™ microbiotests are a 24-h acute lethal toxicity test using larvae (24 h old) of the crustacean T. platyurus and A. franciskana hatched from dormant eggs. The assays were performed in 6 × 4 multi-well plates with five toxicant concentrations and in three replicates, according to the standard operational procedure attached to this microbiotest [47,48]. A positive control using potassium dichromate was conducted in order to verify the sensitivity of the crustaceans (24 h LC_50_: 0.092 mg dm^−3^). The lethal responses of the crustaceans were observed after 24 h of incubation at 25 °C and 24 h LC_50_ values were calculated with the graphical interpolation method using the Regtox-ev6.xls spreadsheet version 6 [35]. Ten crustaceans in each concentration and control were exposed. Immobile crustaceans were considered as those that would not swim within 15 s after gentle agitation. Crustaceans were not fed during acute exposure. A positive control using potassium dichromate was conducted in order to verify the sensitivity of the crustaceans, and solvent controls were run concurrently with all of the tests. 

Statistical analyses of the results were performed using Student’s test (=0.05) in Statistica 13.1 software.

## 4. Conclusions

The present study suggests that magnetite is a highly effective photocatalyst to remove TBBPA from aqueous solutions. The studied TBBPA degradation systems showed photocatalysis to be more effective than ozonolysis (k_app_ was 243.2 × 10^−3^, 272.1 × 10^−3^, and 37.7 × 10^−3^ min^−1^ for μ-Fe_3_O_4_, n-Fe_3_O_4_, and ozonolysis, respectively). The observed removal efficiency for both catalysts (μ-Fe_3_O_4_ and n-Fe_3_O_4_) was comparable. The ozonolysis method of TBBPA removal was relatively low. The best TOC removal was observed for reaction system 1 (m-Fe_3_O_4_), whereas the TOC removal for other systems was comparable. Considering the obtained results, it can be concluded that the best efficiency for the removal and degradation of TBBPA was in reaction system 1 (μ-Fe_3_O_4_ photocatalysis).

This research has demonstrated that the TBBPA solution subjected to the photocatalysis was less toxic to A. franciscana. Debromination of TBBPA is suspected for the production derivatives (BrO_3_^−^) with a high toxicity.

Acute toxicity results show noticeable differences in the toxicity of TBBPA and its degradation products to A. franciscana and T. platyurus. The EC_50_ values indicate that TBBPA degradation products were toxic to harmful, whereas the TBPPA and post-reaction mixtures were toxic to the invertebrate species tested. DMSO, used as a co-solvent, facilitates the transport of the examined organic compounds into the cells of the tested crustaceans, increasing their toxicity.

Our results showed that A. franciscana and T. platyurus are sensitive test animals for the acute toxicity bioassays with the studied compounds.

## Figures and Tables

**Table 1:** Summary of reaction systems.

Reaction System Number	Degradation Process
1	μ-Fe_3_O_4_ photocatalysis
2	n-Fe_3_O_4_ photocatalysis
3	ozonolysis

**Table 2:** EC_50_ levels of tested compounds.

Compound	EC_50_ (24 h), *Artemia franciscana* [mg dm^−3^]	EC_50_ (24 h), *Thamnocephalus platyurus* [mg dm^−3^]
TBBPA (I)	6.243 (4.931–6.772)	4.141 (3.47–4.61)
BPA (II)	34.74 (28.35–43.26)	2.35 (2.03–3.04)
2,6-dibromo-4-hydroxybenzoic acid (III)	34.89 (31.15–41.53)	22.57 (19.44–28.76)
2,6-dibromo-4-isopropylphenol (IV)	34.01 (30.31–39.58)	2.88 (2.64–3.23)
2,6-dibromo-4-methylphenol (V)	35.56 (28.08–39.43)	12.15 (11.54–15.79)
TBBPA/μ-Fe_3_O_4_ (1)	5.56 (4.61–6.25)	4.748 (4.31–5.34)
TBBPA/n-Fe_3_O_4_ (2)	10.95 (10.40–13.36)	2.884 (2.24–3.14)
TBBPA/O_3_ (3)	7.048 (6.14–7.82)	4.436 (4.01–5.65)

**Table 3:** Toxicity of TBBPA and its by-products based on European Union Commission Guideline 93/67/EEC classification [36] and estimated EC_50_ values.

**Compound/Removal System**	**Extremely Toxic** **(<0.1 mg dm^−3^)**	**Very Toxic** **(0.1–1 mg dm^−3^)**	**Toxic** **(1–10 mg dm^−3^)**	**Harmful** **(10–100 mg dm^−3^)**	**Non Toxic** **(>100 mg dm^−3^)**
TBBPA (I)			+ •		
BPA (II)			•	+	
2,6-dibromo-4-hydroxybenzoic acid (III)				+ •	
2,6-dibromo-4-isopropylphenol (IV)			•	+	
2,6-dibromo-4-methylphenol (V)				+ •	
TBBPA/μ-Fe_3_O_4_ (1)			+ •		
TBBPA/n-Fe_3_O_4_ (2)			•	+	
TBBPA/O_3_ (3)			+ •		

+—*A. franciscana.* •—*T. platyurus.*

## Data Availability

Data available upon request due to restrictions, e.g., privacy or ethical. The data presented in this study are available upon request from the corresponding author.
